# Self-management and HeAlth Promotion in Early-stage dementia with e-learning for carers (SHAPE): study protocol for a multi-centre randomised controlled trial

**DOI:** 10.1186/s12889-020-09590-9

**Published:** 2020-10-09

**Authors:** Ingelin Testad, Linda Clare, Kaarin Anstey, Geir Selbæk, Guro Hanevold Bjørkløf, Catherine Henderson, Ingvild Dalen, Martha Therese Gjestsen, Shelley Rhodes, Janne Røsvik, Jessica Bollen, Jessica Amos, Martine Marie Kajander, Lynne Quinn, Martin Knapp

**Affiliations:** 1 Centre for Age-related Medicine – SESAM, Stavanger University Hospital, Stavanger, Norway; 2 University of Exeter, College of Medicine and Health, University of Exeter, Exeter, UK; 3 Department of Old Age Psychiatry, Institute of Psychiatry, Psychology, & Neuroscience, King’s College London, London, UK; 4 REACH: The Centre for Research in Ageing and Cognitive Health, University of Exeter, St Luke’s Campus, Exeter, UK; 5 UNSW Ageing Futures Institute, University of New South Wales, Randwick, Australia; 6 Neuroscience Research Australia, Randwick, Australia; 7 Norwegian National Advisory Unit on Ageing and Health, Vestfold Hospital Trust, Tønsberg, Norway; 8 Department of Geriatric Medicine, Oslo University Hospital, Oslo, Norway; 9 Faculty of Medicine, University of Oslo, Oslo, Norway; 10 Care Policy and Evaluation Centre, Department of Health Policy, London School of Economics and Political Science, London, UK; 11 Department of Research, Section of Biostatistics, Stavanger University Hospital, Stavanger, Norway; 12 Department of Clinical Medicine, University of Bergen, Bergen, Norway

**Keywords:** Self-management, Health promotion, Dementia, Intervention, Group intervention, E-learning, Carers, Self-efficacy, Cost-effectiveness, Randomised controlled trial

## Abstract

**Background:**

With an increasing number of people with dementia worldwide and limited advancement in medical treatments, the call for new and cost-effective approaches is crucial. The utility of self-management has been proven in certain chronic conditions. However, very little work has been undertaken regarding self-management in people with dementia.

**Methods:**

The SHAPE trial will include 372 people with mild to moderate dementia to evaluate the effectiveness and cost-effectiveness of an educational programme combining approaches of self-management, health promotion, and e-learning for care partners. The study is a multi-site, single-randomised, controlled, single-blinded trial with parallel arms. The intervention arm is compared with treatment as usual. The intervention comprises a 10-week course delivered as group sessions for the participants with dementia. The sessions are designed to develop self-management skills and to provide information on the nature of the condition and the development of healthy behaviours in a supportive learning environment. An e-learning course will be provided for care partners which covers similar and complementary material to that discussed in the group sessions for the participant with dementia.

**Discussion:**

This trial will explore the effect of the SHAPE group intervention on people with mild to moderate dementia in terms of self-efficacy and improvement in key health and mental health outcomes and cost-effectiveness, along with carer stress and knowledge of dementia.

**Trial registration:**

ClinicalTrials.gov Identifier: NCT04286139, registered prospectively February 26, 2020, https://clinicaltrials.gov/ct2/show/NCT04286139

## Background

The recent Lancet Commission report on dementia estimated that 47 million people were living with dementia in 2017 [1]. As the world’s population grows older, this number is expected to double every 20 years [2]. With limited advancement in medical treatments combined with increasing pressures on limited resources, novel and cost-effective approaches are needed to reduce the impact of the disease at the individual and societal levels. More effective self-management is central to achieving this objective and has the potential to avoid unnecessary excess disability or premature loss of function and institutionalisation [3, 4]. There is a growing body of evidence for the health and economic benefits of other interventions for people with dementia [5] and for their care partners [6], but so far, little has been done to increase knowledge on the clinical and economic case for self-management, health promotion and e-learning. A number of countries have developed national strategies for dementia and emphasised the importance of providing disease-related information for the person affected by the disease and not just for the care partner [7, 8]. Health plans, in countries including Norway [9] and United Kingdom (UK) [10], are responding to the health needs of individuals with dementia and their families, as well as identifying better ways to care for people with dementia. These policies [11] aim to help citizens stay in their homes for longer, based in part on an economic motivation to address the total direct and formal care cost of dementia, particularly because admission to 24-h care is expensive [12]. Despite the importance of self-management, very little work has been undertaken on either health promotion or self-management in people with dementia.

A recent systematic review [13] focused on self-management for people with dementia and mild cognitive impairment (MCI), and outlined the key components of self-management interventions. It found that while some interventions included some key components of self-management, only two studies specifically reported self-management programmes, and neither of these presented quantitative outcomes. The SMART trial, a pilot randomised controlled trial (RCT) [14] showed quantitative positive findings regarding self-efficacy, and showed high satisfaction as reported qualitatively. Another RCT of self-management [15] also demonstrated beneficial effects on the health-related quality of life of spouses and the cognitive function of persons with dementia. Benefits of self-management include improved knowledge, task performance, self-efficacy and aspects of health status [16, 17]. Most empirical work has focused on the self-efficacy component of this model. Self-efficacy is defined as belief in one’s ability to succeed or to accomplish a specific action in a particular situation [18]. The utility of self-management is long proven for outcomes of other chronic diseases [19, 20]. We have found that people with early-stage dementia are usually able to identify areas of their lives, such as exercise, social activities and recreational pursuits, which they would like to manage better [21, 22], which in turn could improve quality of life and well-being. This offers an avenue for a sensitive and tailored approach to encourage individuals receiving a diagnosis of dementia to draw on their resources and on support from others to make positive changes. Health promotion includes self-management components such as developing personal skills; but also covers a wider set of approaches such as public policy, supportive environments, community actions and health service perspectives [23].

Evidence exists for the need to assist persons with early-stage dementia with health promotion to sustain their function, quality of life, well-being, and prevent premature loss of function or institutionalisation [24]. Buettner and Fitzsimmons [4] demonstrated significant positive change in cognition and depression in their health promotion intervention. The intervention has been adapted and applied to a Norwegian context [25], where a quasi-experimental study including 108 persons with early-stage dementia was conducted. Findings from this study showed a significant improvement in depressive symptoms and self-rated health in early stage dementia, and participants’ MMSE scores stayed stable during the 4-month follow-up, which was statistically significantly different from the average decline in MMSE scores over the same time period among people with dementia [26, 27]. Furthermore, a similar study by Richeson et al. [28] found evidence that health promotion leads to improved self-efficacy in people with early-stage dementia. Other studies of health promotion for people with early-stage dementia have reported improved nutrition, health related quality of life, prevention of falls [29], and improvement in physical function [30]. A systematic review by Boots et al. [31] found beneficial effects of internet-based interventions for care partners of people with dementia. The review demonstrated improvement in various aspects of carer wellbeing, e.g. confidence, depression and self-efficacy, given that the programs include multiple components and are tailored to the individual.

### Study objectives

The primary objective is to determine whether the integrated 10-week SHAPE intervention, combining self-management and health promotion for people with dementia and e-learning for care partners, will significantly improve self–efficacy in people with mild to moderate dementia. Secondary objectives are to determine the impact of the SHAPE intervention on mood, well-being, quality of life, health behaviours, cognition, neuropsychiatric symptoms, carer stress, knowledge of dementia, and to assess cost-effectiveness in people with dementia and their care partners. Furthermore, we wish to qualitatively explore and describe barriers and promoting factors for uptake and implementation, and capture motivational and empowering elements of the intervention and to explore the intervention’s impact on self-efficacy and wellbeing.

### Hypotheses

We hypothesise that the intervention will significantly improve self-efficacy in people with dementia compared to treatment as usual. Secondary hypotheses are that the integrated SHAPE intervention will improve wellbeing and other key health and mental health outcomes for people with dementia (e.g. depression, anxiety, health-promoting behaviour, and quality of life), reduce stress and increase knowledge of dementia for care partners, and provide a cost-effective approach to improving outcomes for people with dementia and their families.

### Assessment and management of risk

This is a low-risk, non-pharmacological trial [32]; in such trials, certain adverse events are not required to be reported, but should be recorded. Minimal risk of psychological stress may occur as a result of assessments (e.g. completing a depression scale). Should psychological distress occur, the research team will provide participants with information about local resources that will assist with their distress.

If a participant is hospitalised during the intervention, this will be recorded. No deaths are anticipated as a result of the intervention. However, if a participant dies during the trial period, follow-up assessments that include the care partner will be discontinued.

## Methods/design

This protocol is reported with reference to the SPIRIT checklist [33] (Additional file 1).

### Overall design

This is a multi-site, controlled, single block-randomised, single-blinded trial with parallel arms. The intervention arm is compared with treatment as usual (TAU).

### The SHAPE intervention

The SHAPE intervention is based on the Corbin and Strauss Trajectory Model [34], and combines approaches of self-management, health promotion, and e-learning. The intervention is designed to develop self-management skills in areas including decision-making, symptom management and social interaction and to provide information on the disease process and the development of healthy behaviours in a supportive learning environment. The intervention comprises ten weekly 2-h group sessions for persons with dementia, where each session is led by two trained facilitators at each study site. The group facilitators will have professional clinical training e.g. nursing, occupational therapy, and psychology and have experience of working with people with dementia. For the care partners involved as study partners, the same material presented in the 10-week SHAPE course for people with dementia, plus some additional material and signposting to support them in their role, will be delivered through an online e-learning platform. Key themes of the intervention will include positive actions to improve and maintain health, how to talk about the impact of the disease on the life of the person with dementia, addressing the fear of losing independence and how to tackle and solve other sensitive issues (Table 1).

**Table 1:** Key themes covered in the intervention

Sessions	Topic
Session 1	Orientation to the course
Session 2	Healthy lifestyle, doctor’s visits and use of medication
Session 3	Healthy eating, diet and nutrition
Session 4	Staying safe
Session 5	Exercising body and brain
Session 6	Activities, interests and learning
Session 7	Managing memory difficulties
Session 8	Adapting and coping
Session 9	Relationships and communication
Session 10	Planning for the future

### Control arm

**Fig. 1:**
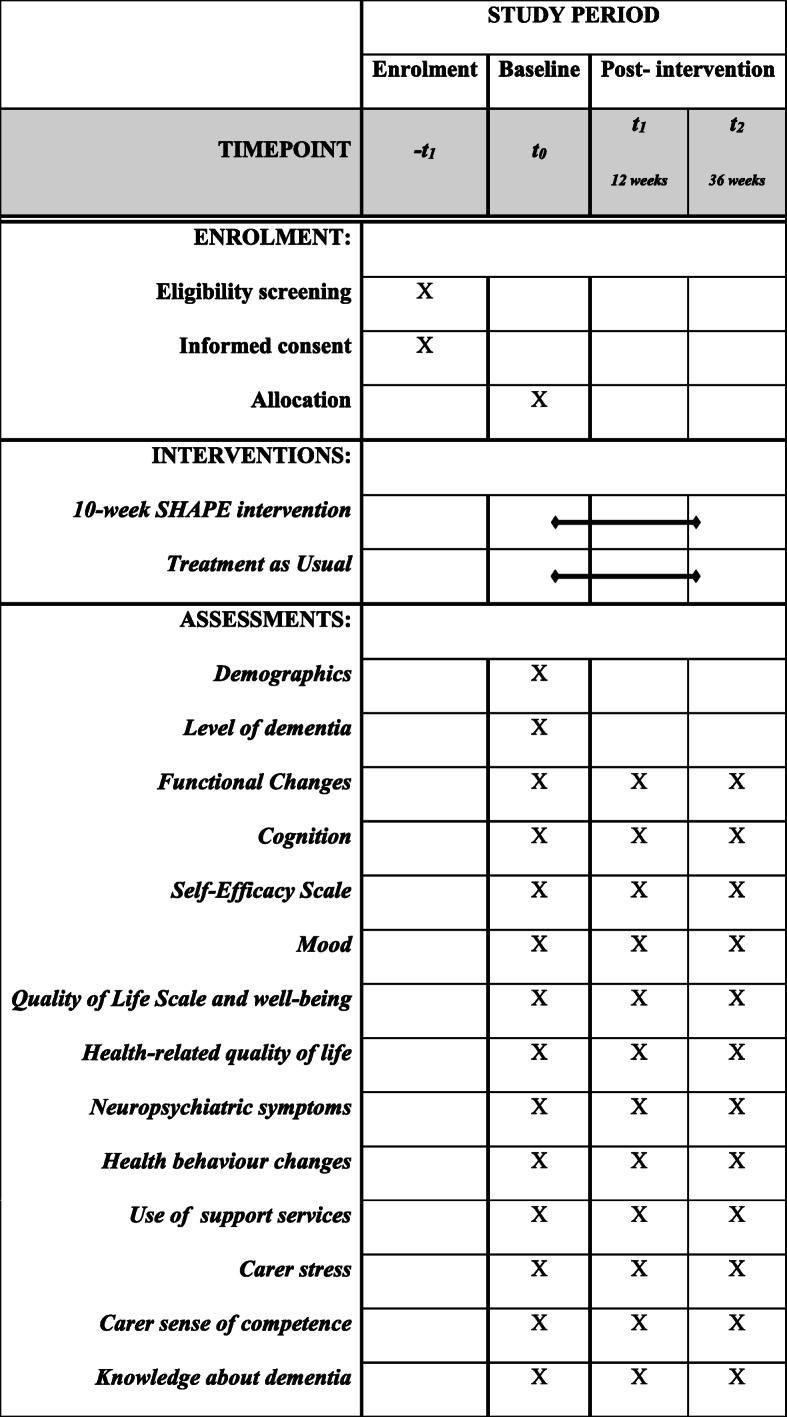
Standard protocol items recommendations for intervention trials (SPIRIT): Schedule of enrolment, intervention, and assessments for the SHAPE trial

Participants randomised to TAU will continue to receive usual care and support, which may include a regular nurse-led clinical review and access to services such as psychiatry, occupational therapy and social services as needed. Using TAU as a comparator condition ensures that all participants receive needed services and allows for a comparison between the new intervention and current best practice. However, after the intervention is completed, the TAU group will be offered the intervention. Furthermore, all participants in the TAU group will be given the link to the e-learning comprising the complete educational programme of the intervention once the trial has ended. Schedule of enrolment, intervention, and assessments for the SHAPE trial are presented in Fig. 1.

### Sample size

The sample size calculation is based on the approach outlined in Lohr et al. [35] and the aim of detecting a standardised mean difference of 0.4 in the General Self-Efficacy Scale (GSES) between the intervention and control arms of the trial, with 90% power at the 5% significance level. An effect size (Cohen’s D) of 0.4 is a commonly used threshold for clinical meaningfulness [36]. A standard sample size calculation suggests that 268 participants would be required (134 participants per arm). Including in the analysis covariates that are predictive of the outcome will increase the precision of the effect estimate and hence the statistical power. Assuming a coefficient of determination R2 of 0.25 for the regression of the outcome on baseline variables, the required total sample size is reduced to 201 [37].

Due to the potential for a clustering effect in the intervention because participants are attending group sessions where the outcomes for individuals nested within the same educational groups [WS8] are likely to be more similar than the outcomes for individuals within different groups, the variance in the intervention arm will be inflated. We assume a residual intra-cluster correlation coefficient (ICC) of 0.1. We plan for group sizes of 8 in the intervention arm and an allocation ratio of 2:1 (i.e. 67% of participants will be allocated to the treatment arm). Assuming 25% attrition we anticipate an average number of 6 participants within each cluster contributing data for analysis. Given this plan, and allowing for attrition, we will need to recruit a total of 246 participants to the intervention arm and 123 participants to the TAU arm in order to provide sufficient power for the primary analysis. Harmonising to a fixed block size we will recruit 248 participants to the treatment arm and 124 participants to the control arm, i.e. a total of 372 participants. Additional allowance for varying group sizes is not necessary if the coefficient of variation in cluster sizes is smaller than 0.23 [38], which we expect to be the case in this study design.

### Recruiting centres

Stavanger University Hospital (SUH) and Norwegian National Advisory Unit on Ageing and Health in Norway (AH), the University of Exeter and partner National Health Service (NHS) Trust(s) in the UK, the University of New South Wales with Prince of Wales Hospital (UNSW) and Dementia Australia as partners will all act as recruiting centres.

### Planned recruitment rate

Across all four sites combined, we will recruit a total of 372 participants to the study. The recruitment will happen in blocks of 12 participants at each site, of which 8 will be randomised to the SHAPE intervention and 4 to TAU. The planned distribution among the centres is 84 participants at Exeter, 96 at A&H, 108 at SUH and 84 at UNSW.

Month 1 will be spent recruiting the first blocks of participants, who will have their baseline visits in the first 2 weeks of month 2, with a maximum of 2 baseline interviews per day at each centre. Following this we will randomise consenting participants, and inform them of their allocation and for those in the intervention arm the start date of the course. Start-up of the first course groups will be in month 3. Further recruitment will happen concurrently, and we anticipate that we will have enough participants to start up at least one new course group at each centre every 1.5 months, meaning that recruitment will be completed in 13 months, and the last course groups will start in month 15.

### Randomisation

When recruitment reaches a group target of 12 consented pairs of participant and carer partners at a site we will proceed to randomisation. A computer-generated randomisation sequence will be used to assign the participant pairs in each site to the intervention and control arms. A block randomisation scheme will be implemented to ensure an appropriate ratio in the number of participant pairs allocated to each trial arm, stratified by group delivery site. The allocation sequence will be concealed from researchers (outcome assessors) using an online central randomisation service set up and maintained by the Exeter Clinical Trials Unit (UKCRC Registration ID 65). Group facilitators and pairs of participant and care partners will not be blinded.

The participants and their care partners will receive an email and letter indicating the result of randomisation. Participants allocated to the intervention arm will be sent details of the group sessions and will be contacted by their Lead Facilitator before the first group session, and their care partner will receive a link to the online e-learning platform.

### Blinding

Quantitative data will be collected by a researcher blind to the participants’ allocation (intervention vs TAU).

### Outcome and study eligibility measures

All quantitative outcome measures will be assessed prior to randomisation (baseline), after the intervention (follow-up 1) and then 6 months after follow-up 1 (follow-up 2). The assessments will consist of questionnaires using standardised, evidence-based scales and other information obtained from the participant and appointed care partner. The interviews will be conducted with the participant and care partner, both together and separately. Participant and care partner demographic details will be collected.

The primary outcome in the SHAPE trial is self-efficacy measured by the General Self-Efficacy Scale (GSES) [39]. The secondary outcome measures include cognition assessed with the Mini Mental State Examination (MMSE) [40], which is also used to check eligibility, depressive symptoms measured by the Cornell Scale for Depression in Dementia (CSDD) [41], quality of life and well-being measured by the Dementia Quality of Life Scale (DEMQOL) [42], health-related quality of life measured by EQ-5D-5L [43], neuropsychiatric symptoms measured by the Neuropsychiatric Inventory Questionnaire (NPI-Q) [44], health behaviour changes measured by the SHAPE research team health reporting form, use of support services measured by the Client Services Receipt Inventory (CSRI) [45], carer stress measured by the Relative‘s Stress Scale (RSS) [48], carer sense of competence measured by the Short Sense of Competence Questionnaire (SSCQ) Short version [49], and knowledge about dementia measured by the Dementia Knowledge Assessment Scale (DKAS) [50]. We will use the Clinical Dementia Rating Scale (CDR) [39] to measure level of dementia and to measure functional changes in the participants we will use the Functional Activities Questionnaire (FAQ) [40]. A full description of the primary and secondary outcomes and screening instruments is provided in Table 2.

**Table 2:** Description of measures

Measures	What does the tool measure	Informant	Tool characteristics and psychometric properties
CDR [49]	Diagnostic criteria	Care partner	Assesses six domains of cognitive and functional performance: memory, orientation, judgment & problem solving, community affairs, home & hobbies, and personal care. 0 = no cognitive impairment, 0.5 = questionable or very mild dementia, 1, 2 and 3 for mild, moderate and severe dementia. Scores in each of these are combined to obtain a composite score ranging from 0 (none) through 3 (severe).
FAQ [50]	Functional changes	Care partner	10-item scale measuring instrumental activities of daily living. The score range for each item is 0–3, with higher scores indicate greater impairment; 0 = normal or never did but could do now; 1 = has difficulty but does by self or never did but would have difficulty now; 2 = requires assistance; 3 = dependent.
MMSE [40]	Cognition	Participant	30-point questionnaire, with items assessing orientation, attention, immediate and short-term recall, language, and the ability to follow simple verbal and written commands. Lower scores indicating more severe cognitive problems.
GSES [39]	Self-efficacy *Primary outcome*	Participant	10-item psychometric scale designed to assess a person’s sense of competence for dealing effectively with a variety of stressful situations. Responses are rated on a 4-point Likert scale. Total score ranges from 1o to 40, with higher values indicating greater self-efficacy.
CSDD [41]	Depressive symptoms	Participant and care partner separately	Based on impressions from interviews with the person with dementia and their care partner, the final ratings of the CSDD items represent the rater’s clinical impression rather than the responses of the care partner or the person with dementia. The scale consists of 19 items that ranges from 0 (absent) to 2 (severe). Total score ranges from 0 to 38, with higher values indicating more depressive symptoms.
DEMQOL [42]	Quality of life and well-being	Participant and care partner separately	The measure consists of two questionnaires. DEMQOL, conducted with person with dementia is a 28-item interviewer- administered questionnaire with the score range of 28 to 112. DEMQOL-Proxy is a 31-item interviewer-administered questionnaire answered by a care partner with a score range of 31 to 124. The DEMQOL-Proxy is also validated as a method for calculating QALYs for health economic analysis.
EQ-5D-5L [43]	Health-related quality of life	Participant and care partner separately	Participant and their care partner indicate the participant’s health state across five dimensions: mobility, self-care, usual activities, pain/discomfort and anxiety/depression. Each dimension has 5 levels: no problems, slight problems, moderate problems, severe problems and extreme problems. Additionally, the participant’s self-rated health is measured on a vertical, visual analogue scale, where 0 represents ‘worst imaginable health state’ and 100 represents ‘best imaginable health state’.
NPI-Q [44]	Neuropsychiatric symptoms	Care partner	12-item questionnaire developed to assess behavioural disturbances in people with dementia. NPI-Q is a validated structured interview assessment with a care partner. Scores are entered for the frequency and severity of each symptom over the last four weeks, and subsequently multiplied into a symptom score. The total possible maximum score is 144. A higher score reflects increased frequency and severity of the disturbances.
Self-rated Health Behaviour Change	Health behaviour changes	Participant and care partner separately	The SHAPE research team will create a health reporting form based on the SHAPE intervention that asks about specific health-related change that occurred during each time period. Data from self- and family report.
CSRI [45]	Use of support services	Participant and care partner together	Used to estimate the cost of the participant’s service package and of support services for the care partner. Information collected: participant’s use of hospital, community-based and day services, participant’s mental health medications, care partner’s use of support services and mental health medications. Questions cover a retrospective period of 3 months.
RSS [46]	Carer stress	Care partner	15-item, 5-scale self-report measure designed to assess the degree of distress and social upset experienced by a relative as the result of caring for a person with physical and/or behavioural difficulties.
SSCQ [47]	Carer sense of competence	Care partner	7-item questionnaire covering 3 domains: consequences of involvement in care for the personal life of the care partner, satisfaction with one’s own performance as a care partner, and satisfaction with the person with dementia as a recipient of care.
DKAS [48]	Knowledge about dementia	Care partner	25-item scale measuring dementia knowledge across four domains: causes and characteristics, communication and behaviour, care considerations, and risks and health promotion.

CDR: Clinical Dementia Rating Scale, FAQ: Functional Activities Questionnaire, MMSE: Mini Mental State Examination, GSES: General Self-Efficacy Scale, CSDD: Cornell Scale for Depression in Dementia, DEMQOL: Dementia Quality of Life Scale, NPI-Q: Neuropsychiatric Inventory Questionnaire, CSRI: Client Service Receipt Inventory: RSS: Relative‘s Stress Scale, SSCQ: Short Sense of Competence Questionnaire, DKAS: Dementia Knowledge Assessment Scale

### Economic evaluation

For the economic evaluation, there will be two co-primary outcomes: self-efficacy (GSES) [39] and QALY gain (calculated from EQ-5D-5L self-report; societal weights) [43]. Secondary outcomes will be QALY gain from DEMQOL self-report [51], care partners’ QALY gain (calculated from carer-reported EQ-5D-5L), CSDD [41], and carer stress (RSS) [46]. Two perspectives will be employed: health and social care system, and societal (including costs of unpaid care).

Costs of the self-management intervention (e.g. staff costs, room hire and materials) will be collected on pro-formas devised for the study; costs of the e-learning course for care partners will be collected from the project team. We will calculate incremental cost-effectiveness ratios (ICER) as the difference in costs between SHAPE and TAU (either service-related or societal, depending on perspective) over the difference in outcomes between groups, for each outcome in turn. Service-related costs will be based on (i) participant-with-dementia service costs; and (ii) costs of participants’ and care partners’ service use. In addition, an ICER will be calculated from participant-plus-carer costs and combined participant and care partner QALY (calculated from EQ-5D). Cost-effectiveness analyses will be conducted using a combination of bootstrap sampling and cost and outcome regressions taking into account clustering, correlations between costs and outcomes, and skewed dependent variables. Regressions will include treatment allocation; baseline scores of the dependent variable (e.g. utility, GSES, costs and other baseline variables as relevant). Sensitivity analyses will explore impact on findings of changes in key cost and outcome assumptions. We will calculate net monetary benefit and construct cost-effectiveness acceptability curves to explore the likelihood of cost-effectiveness over a range of willingness-to-pay values, taking into account decision and sampling uncertainty [52, 53].

### Qualitative evaluation

Qualitative outcome will be obtained during the intervention and immediately after group completion, using direct observations of participants and focus groups with group facilitators. The qualitative evaluation is designed to develop a deeper understanding of and insight into the intervention to i) provide recommendations for future work and ii) explore the impact of the intervention on self-efficacy and wellbeing.

#### Group observations

To study the process of engagement in the group sessions, we will observe 5 groups (3 in Norway, 1 in UK and 1 in AU), each consisting of up to 8 participants (*n* = 40) over the duration of the course (10 weeks). We will use a moderate participant observation method, where the researchers are present and identifiable, without engaging in any form of interaction with the participants [54]. The observations will be carried out on study commencement, by an observation team with a minimum of two researchers with different backgrounds. We will develop an observational protocol that includes a range of pre-selected topics, including (1) in-session behaviour, (2) participant engagement, (3) social interaction within the group, (4) peer support and (5) change in perceptions of living with dementia. The field notes will be transferred to word documents for analysis.

#### Focus groups

To identify promoting and hindering factors affecting the SHAPE intervention, focus groups involving the group facilitators (*n* = 4–8) will be conducted in all sites (*n* = 5, i.e. 3 in Norway, 1 in UK and 1 in AU). The focus groups will be led by an experienced researcher (moderator), and a co- moderator will make notes on observations and impressions during the focus group discussions. A semi-structured topic guide, which will be revised iteratively allowing the main issues identified by participants to be explored in depth, will guide the discussions (see Additional file 1). The focus groups will be recorded and transcribed verbatim; observations and impressions will be noted at the end of each group.

### Intervention fidelity

To monitor intervention fidelity, group facilitators will complete checklists of topics covered in each session and outline any deviations from the session plans. Participants’ attendance at the sessions will be recorded. If participants do not meet up for a session, the group facilitators will follow-up with a phone call.

### Participant selection: inclusion, exclusion and withdrawal criteria

#### Inclusion criteria

- Diagnosis of dementia according to the ICD-10 classification [55] or the Diagnostic and Statistical Manual of Mental Disorders (DSM) IV or V [56]
- 65 years of age or older
- Mild to moderate dementia, defined as MMSE ≥18
- Ability to read and write
- Hearing and vision that are sufficiently good to work in a group setting
- Capacity to give consent for participation in the study
- Proficient in the language in which the intervention is provided
- Care partner willing to participate

#### Exclusion criteria

- A diagnosis of alcohol or drug abuse
- Lewy body dementia, Fronto-temporal lobar degeneration or Semantic dementia
- A limited life expectancy due to any terminal disease or other serious illness, other than dementia
- Chemotherapy or radiation treatment ongoing at enrolment
- Currently participating in another health promotion or self-management group

### Withdrawal criteria

Individual participants can withdraw from the study at any time, without giving a reason.

### Enrolment procedure

**Fig. 2:**
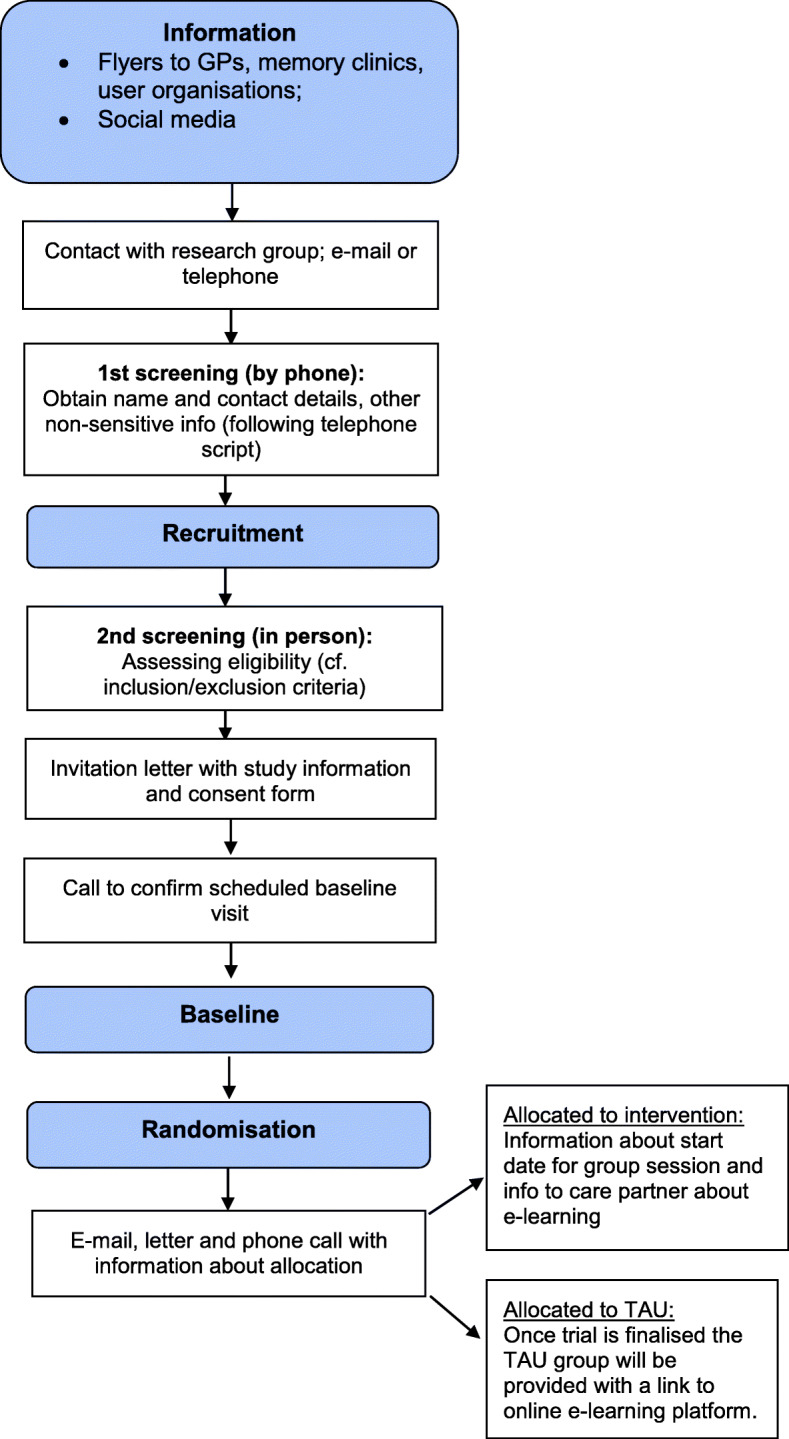
Enrolment process

Details of enrolment, how the participants will be informed whether they have been allocated into the intervention or TAU, and enrolment in the e-learning program are shown in Fig. 2. Information will be given to primary care services, including general practitioners (GP) offices; memory clinics, user organisations and social media through flyers in the areas of the individual study sites.

### Safety reports of serious adverse events (SAE)

Fatal and life-threatening events (otherwise known as Serious Adverse Events) experienced by participants while participating in the study are unlikely to be caused by participation in this trial; however, they will be recorded. SAE reporting will be done according to the requirements of the National Research Ethics Service (NRES) in each individual country. The site PI will inform trial managers and the sponsor of local procedures and ensure that these are followed at each site. SAEs will also be reported to the sponsor and Independent Data Monitoring Committee (IDMC) every 6 months, unless more frequent reporting is requested. PIs will also ensure that AEs are recorded, but there is no requirement to report these to the sponsor or regulatory bodies unless requested.

The IDMC will consist of an independent statistician (Chair), a consumer/user representative and a researcher. There will also be a trial representative on the committee. The IDMC will meet every 6 months and if necessary in response to any serious untoward incidents. The IDMC will be responsible for monitoring serious adverse effects, protocol violations and any risks emerging from the trial.

### Data management and analysis

It is planned that anonymous data and all appropriate documentation will be kept securely for the defined period required from the relevant ethics committees. Participants will be clearly informed that their data will be used for the stated purposes of the study. Only members of the study team and its support staff will see the data. A full clinical Data Management Plan will be written by the trial team before recruitment commences, which will include a privacy impact assessment. All paper-based data will be double entered. Data will be entered into a secure Electronic Data Capture System (EDC). This database is built to validated standards and maintained and managed by the UKCRC registered Exeter Clinical Trial Unit.

Data from questionnaires will be anonymised through the use of unique participant identification codes when entered into computers for statistical analysis.

Qualitative data from the interviews will be transcribed verbatim and then anonymised through the removal of people’s names and other personal information; where necessary non-identifiable terms or pseudonyms will be used instead. In reports of the work, where excerpts are quoted from interviews, any information that might lead to the identity of participants, other people or organisations being inferred will be disguised.

All data will be collected and stored in accordance with data protection regulations in the individual countries. Data will be stored electronically on computers and access will be controlled via passwords and permissions to dedicated study folders. Hard copies of questionnaires will be securely stored in locked filing cabinets in offices that are accessible only to research staff. Information used in the administration of the study, including participants’ names and addresses, will be stored separately from the research data and used only to maintain contact with participants. Administrative databases will be held at the study centres.

The CI will preserve the confidentiality of participants taking part in the study and is registered under the EU GDPR (General Data Protection Regulation (Regulation (EU) 2016/679)). The research will follow GDPR guidance. Only members of the research team will have access to the original data, which will be stored in a locked filing cabinet. Participants’ personal details will be stored separately from the original data, and will be kept in a separate file on a password protected computer at the study sites. Access to data will be limited to quality control, audit, and analyses. Data shared between sponsor and co-investigators will be de-identified to minimise breach of confidentiality. Each participant will be assigned an identification code, which will be used in all data storage files; these will not contain names or any other means of personal identification. All personal details will be deleted on completion of the study.

### Statistical analysis

All outcome measures will be reported at baseline, after the intervention (follow up 1) and at 6-months after follow-up 1 (follow up 2). Baseline characteristics (age, gender, ethnicity, civil status, education, living situation, use of medication) will be presented for each intervention group and by site. Trial analysis will include all participants with allocation to study arms as randomised, i.e. on an intention-to-treat basis.

The primary outcome measure (GSES) [39] will be analysed using the multilevel modelling approach to analysis of covariance (ANCOVA), i.e. with follow-up GSES as dependent variable and with study arm, site Norway 1, Norway 2, UK and Australia, baseline GSES, time since baseline assessment, other baseline variables predictive of the outcome, as well as potential variables constituting baseline imbalance between study arms, as independent variables, while allowing for clustering within treatment groups [57]. The estimate of the effect of the intervention will be reported with a 95% confidence interval and associated *p*-value. Missing outcome data will be handled by including any variables predictive of missingness in a complete case-analysis of the multilevel ANCOVA model [58]. The estimate of the effect of the intervention will be reported with a 95% confidence interval and associated *p*-value. Secondary outcomes will be analysed similarly.

Multiple imputation (MICE) will be used for sensitivity analysis to assess the robustness of the results to differential loss to follow up [58].

### Qualitative analysis

Qualitative data will be analysed by way of systematic text condensation [59]. This approach involves the following steps in the analysis process: (1) establishing an overall impression of the data material and identifying preliminary themes by reading through the transcripts several times; (2) identifying and sorting units of meaning into code groups; (3) condensing the contents of each of the coded groups into subgroups; and (4) summarising and re-contextualising the contents of each code group to generalise descriptions and concepts. Malterud [59] argues that the data analysis will benefit from being conducted by more than one researcher; thus all transcripts will be read by several members of the research team to get an overall impression of the full data material, as in step (1) above. This step of the analysis requires the researcher to read with an open mind from a bird’s-eye perspective all pages within the transcripts, and then ask which preliminary themes (usually four to eight themes) can be identified in the material.

### User involvement

People with dementia, care partners and group facilitators from earlier health promotion groups [25] will be involved in all levels and all phases of the study including research planning, delivery and dissemination, and there will be extensive use of co-design groups and close collaboration with organisations representing people with dementia. This will ensure that the development and delivery of the integrated intervention for people with dementia and their families is centred around the needs of people with dementia themselves in all of the participating countries. Each site will appoint a user representative as a member of the respective research teams. This will ensure that perspectives from people with dementia, care partners and group facilitators from earlier health promotion groups are included and addressed throughout the trial.

### Study management

This study will be managed by the chief investigator (Ingelin Testad), the trial manager (Martha Therese Gjestsen) and the Programme Management Group (PMG), as part of the overall SHAPE programme. The PMG will be chaired by Lynne Quinn, Clinical Trial Unit, Exeter University and involve all the PIs, the trial manager and a consumer representative. The group will meet at 3 monthly intervals and additionally as required. Part of the remit of the PMG will be to oversee overall progress of the programme and monitor progress against milestones. Any discrepancy from milestones will be highlighted and a plan, developed to address the difficulties, will be instigated. For the purposes of this trial, the PMG will be acting as the Trial Management Group (TMG), with direct oversight of and responsibility for this study. The trial manager will send a written report to the chair of the PMG before each TMG meeting, detailing progress.

The day-to-day management of the trial will be conducted by the trial manager, Martha Gjestsen, who will be supervised (weekly) and line managed by Ingelin Testad. Additional supervision will be organised as needed, and major decisions will be discussed with PI group and PGM group, as appropriate.

### Indemnity

Each participating site will be responsible for providing indemnity to meet the potential legal liability of investigators/collaborators arising from harm to participants in the conduct of the research. No provision is made for non-negligent liability, which will be covered by usual procedures by the care provider as applicable.

### Study sponsor

This study is sponsored by Stavanger University Hospital (SUH), Armauer Hansens vei 2, 4011 Stavanger, Norway. Telephone: + 47 51 51 98 28, svein.skeie@sus.no

### Publication and dissemination strategy

The study’s Dissemination and Publication Policy will be developed by the trial team and approved by the PMG as part of the Publication strategy for the whole SHAPE programme. To ensure authorship eligibility the Vancouver recommendations will be used. Scientific outputs, the use of the networking opportunities afforded by each country and making direct contact with local Commissioners will all ensure dissemination. A full and open publication of the results will be provided through peer-reviewed scientific journals. Additional communication will be undertaken through the web pages of the EU JPND, Stavanger University Hospital, Ageing and health, University of Exeter, Alzheimer’s Society and University of New South Wales.

## Discussion

The integrated SHAPE intervention represents a novel and different approach combining self-management and health promotion for people with dementia and e-learning for care partners. It is based on health promotion and self-management and on the imperative to maintain the dignity and autonomy of the person with dementia and support him or her in planning for the future together with the family. The adjunctive e-learning platform will provide care partners with the same information that the person with dementia receives plus some additional material and signposting to support them in their role. This will further empower the whole family to support and enable more effective self-management by the person with dementia, and enhance their ability to plan ahead together and make key decisions jointly - such as how and when to communicate their needs to health care services and to receive appropriate care at the right time. Being able to talk openly about the disease process and future challenges with family members in the early stage of the disease can be empowering for everyone involved. The website with the e-learning resources provided to the care partners will collect information on website use. The e-learning component will also be created in a version which does not collect any data on the user. The purpose of this is so it can be distributed to other people in the support network of the person with dementia, and it therefore has the potential to reach those who live in regional, rural and remote areas.

### Trial status

At the time of submission of this protocol paper (protocol version 6, dated 10.03.2020) was not open to recruitment. Recruitment is scheduled to start in November 2020, providing the COVID-19 pandemic situation allows this, and to be completed by January 2022.

## Supplementary information

**Additional file 1.** THEMATIC FOCUS GROUP INTERVIEW GUIDE.

## Data Availability

Not applicable.

## Abbreviations

AH: Norwegian national advisory unit on ageing and health
AE: Adverse event
ANCOVA: Analysis of covariance
CDR: Clinical dementia rating
AU: Australia
CI: Chief investigator
CSDD: Cornell scale for depression in dementia
CSRI: Client service receipt inventory
DSM: Diagnostic and statistical manual of mental disorders
DEMQOL: Measure of health related quality of life for people with dementia
EDC: Electronic data capture system
FAQ: Functional activities questionnaire
FU: Follow-up
GCP: Good clinical practice
GDPR: General data protection regulation
GP: General practitioner
GSES: General self-efficacy scale
ICC: Intraclass correlation coefficient
IDMC: Independent data monitoring committee
JPND: EU joint programme for neurodegenerative diseases
MCI: Mild cognitive impairment
MICE: Multiple imputation
MMSE: Mini mental state examination
NHS: National health service
NPI-Q: Neuropsychiatric inventory questionnaire
NRES: National research ethics service
NSW: New South Wales university
PI: Principal investigator
PMG: Programme management group
QALY: Quality adjusted life year
RCT: Randomised controlled trial
RSS: Relative‘s stress scale
SAE: Serious adverse event
SHAPE: Self-management and health promotion in early-stage dementia with E-learning for carers
SUH: Stavanger university hospital
TAU: Treatment as usual
TMG: Trial management group
UK: United Kingdom
UKCRC: UK clinical research collaboration
UNSW: University of New South Wales
