# Supplementary material for: Self-management and HeAlth Promotion in Early-stage dementia with e-learning for carers (SHAPE): study protocol for a multi-centre randomised controlled trial
Source: BMC Public Health. 2020 Oct 9;20:1508. doi: 10.1186/s12889-020-09590-9 (PMC7545375; doi:10.1186/s12889-020-09590-9)
Supplement: Supplementary file 1 — Additional file 1. THEMATIC FOCUS GROUP INTERVIEW GUIDE. [file 12889_2020_9590_MOESM1_ESM.pdf]

THEMATIC FOCUS GROUP INTERVIEW GUIDE

1) Describe hindering and promoting factors to the structure of the SHAPE intervention

Additional to guide and support the discussion if needed:

Describe hindering and promoting factors of the environment

Describe hindering and promoting factors of the framework

Describe hindering and promoting factors of the communication

Describe hindering and promoting factors of the training material

Describe hindering and promoting factors in the group and group management

Describe hindering and promoting factors brought up by the participants concerning the e-learning for carers

2) Describe hindering and promoting factors to the process of the SHAPE intervention

Additional to guide and support the discussion if needed:

Describe hindering and promoting factors to participants' motivation

Describe hindering and promoting factors to active group participation

Describe hindering and promoting factors to exchange of experiences, thoughts and feelings

3) Describe hindering and promoting factors to the result of the SHAPE intervention

Additional to guide and support the discussion if needed:

Did you observe change in health behaviour in the participant?

Did you observe new self-management approaches in the participant?
